# Class III β-Tubulin Counteracts the Ability of Paclitaxel to Inhibit Cell Migration

**DOI:** 10.18632/oncotarget.250

**Published:** 2011-05-16

**Authors:** Anutosh Ganguly, Hailing Yang, Fernando Cabral

**Affiliations:** ^1^ Department of Integrative Biology and Pharmacology, University of Texas Medical School, 6431 Fannin St., Houston, Texas 77030, USA

**Keywords:** tubulin isotypes, microtubules, dynamic instability, motility, drug resistance

## Abstract

Class III β-tubulin (β3) is associated with tumor aggressiveness, resistance to therapy, and patient relapse. To elucidate its action, we tested β3's effect on cell migration. Expression of β3 in HeLa and MCF-7 did not alter the intrinsic rate of cell migration, but it prevented the inhibition of migration by low, nontoxic concentrations of paclitaxel. The effects on cell motility were confirmed in CHO cells with tetracycline regulated expression of β3. Cell migration and microtubule dynamics were inhibited by similar concentrations of paclitaxel, but required a 5-10 fold higher drug concentration when β3 was expressed. The directionality of migration was normal in paclitaxel, but cells spent more time in a “paused” state during which there was no net movement. These studies support a model in which paclitaxel inhibits cell migration by suppressing microtubule dynamics and β3-tubulin counteracts paclitaxel action by maintaining microtubule dynamic activity. The results provide a potential explanation for the aggressiveness of β3-expressing tumors.

## INTRODUCTION

Microtubules form an important cytoskeletal network involved in cell shape, vesicle transport, cell motility, chromosome segregation, and cell division. Cellular microtubules are composed of αβ-tubulin heterodimers that assemble into linear protofilaments that associate laterally to form hollow, tube-like structures. The αβ heterodimers are added in a polarized fashion resulting in asymmetric filaments whose fast growing plus-ends are oriented towards the cell periphery while their slow growing minus-ends remain embedded in the centrosome near the cell center. Both α- and β-tubulin are encoded by multiple genes that are expressed in a tissue specific manner. In the case of β-tubulin, there are at least 7 vertebrate genes that produce distinct isotypes: β1, β2, β3, β4a, β4b, β5, and β6. The β1, β4b, and β5 isotypes are found in most mammalian tissues, whereas β2, β3, and β4a are predominantly found in brain, and β6 is restricted to platelets and megakaryocytes [1, 2].

The functional consequences of expressing different tubulin genes have long been a subject of speculation [3]. Studies in cultured mammalian cells showed that cellular microtubules incorporate all available β-tubulin isotypes including ectopic and chimeric proteins with little or no change to the microtubule network [4-7]. On the other hand, studies in transgenic mice revealed an important role for β6 in platelet function [8]. In our laboratory we recently used tetracycline regulated expression to examine the effects of ectopic β-tubulin cDNAs on cell behavior. Our studies have shown that overexpression of β1, β2, or β4b has no obvious effects on the transfected cells [9], and that β4a overexpression has only subtle effects on microtubule assembly and drug sensitivity [10]. In contrast, overexpression of the more divergent β5 and β6 isotypes produces dramatic effects on cell division, microtubule assembly, and cellular responses to drugs that target the microtubule cytoskeleton [11, 12].

The β3 isotype falls between these extremes. Its expression was reported to be increased in cell lines selected for resistance to paclitaxel [13, 14]. We confirmed its participation in paclitaxel resistance but showed that it could only confer very weak resistance and that it acted by reducing microtubule assembly [15]. Although β3 is normally restricted to neuronal and Sertoli cells, it has been found to be inappropriately expressed in tumor cells from diverse tissues and its presence appears to correlate with tumor aggressiveness and resistance to therapy [16, 17]. As a measure of tumor cell aggressiveness, we tested the effects of β3 expression on the ability of paclitaxel to inhibit cell migration.

## RESULTS

### Neuron specific β3-tubulin is expressed in non-neuronal cancer cell lines

Cell migration is a complex process that involves the microfilament and microtubule cytoskeletal systems [18, 19]. We recently showed that concentrations of microtubule inhibitors that are too low to affect cell division are nevertheless efficient at suppressing microtubule dynamics and inhibiting cell motility [20]. Because β3-tubulin has been reported to inhibit paclitaxel's ability to suppress microtubule dynamics [21], we reasoned that β3 expression in tumor cells might affect the ability of drugs like paclitaxel to inhibit cell motility. This in turn could potentially account, at least in part, for the observation that β3 expressing cells tend to be more aggressive and less susceptible to therapy with microtubule targeted drugs [16, 17].

**Figure 1:**
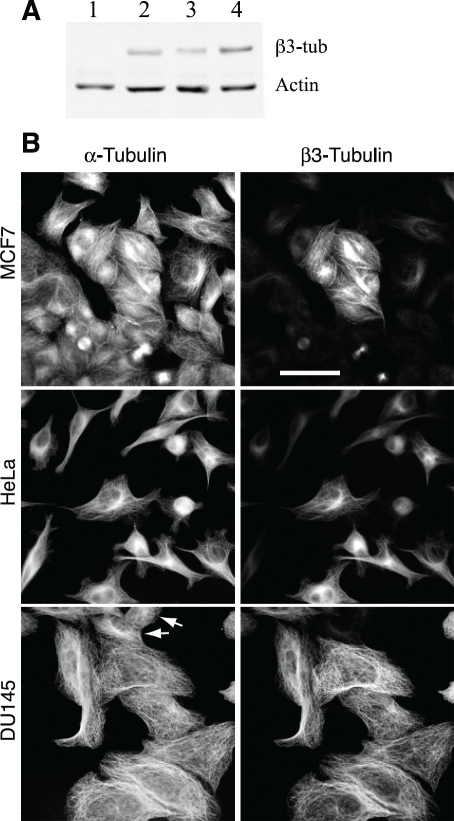
β3-Tubulin production in human cancer cell lines (A) CHO (lane 1), MCF7 (lane 2), HeLa (lane 3), and DU145 (lane 4) cells were lysed and analyzed on western blots with antibodies to β3-tubulin and actin. (B) The indicated cell lines were stained with antibodies specific for α-tubulin and β3-tubulin. Note the heterogeneity of the cells with respect to β3-tubulin staining. Arrows for DU145 indicate the rare presence of cells that are β3 negative. Bar, 50 μm.

To test this hypothesis, we screened several human tumor cell lines for their expression of β3-tubulin using both immunofluorescence and western blot analysis with an antibody specific for the β3 isoform. As a control, we used CHO cells that were previously shown to express β1, β4b, and β5 tubulin, but not β3 [22, 23]. As shown in Figure 1A, CHO cells (lane 1) failed to react with the antibody; but MCF7 (lane 2), HeLa (lanfe 3), and DU145 (lane 4) cells, derived from human breast, cervical, and prostate tumors respectively, showed the presence of varying amounts of β3. Immunofluorescence microscopy (Figure 1B) confirmed the presence of β3-tubulin in the microtubules and further indicated that the staining varied greatly on a cell-to-cell basis. For example, we estimated that only 25% of the cells in the MCF7 cell population were positive for β3; whereas HeLa was 40% positive. Only the DU145 cells appeared mostly uniform for β3 production, but even in that cell line, there were some negative cells (arrows, Figure 1B). We also examined K562 and KB3 cells but found no evidence for β3 production by either western blot analysis or by immunofluorescence microscopy (data not shown). The results confirmed the presence of β3-tubulin in some, but not all, human tumor cell lines.

### Cells that produce β3-tubulin resist the inhibitory effects of paclitaxel on cell migration

We used the heterogeneity of β3 expression in MCF7 and HeLa cell lines as an advantage in order to assess the effects of this isotype on cell motility. Cells were grown on glass coverslips to about 85% confluence, a scratch was made in the monolayer, and the movement of cells into the wound was monitored by time lapse microscopy in the presence and absence of paclitaxel using a concentration that was one fifth of the cytotoxic IC_50_ value. Previous studies indicated that this concentration of drug would be effective at suppressing microtubule dynamics without affecting mitosis [24]. We predicted that in the presence of drug, the migration of cells lacking β3 would be inhibited, but β3-producing cells would continue to migrate and become concentrated at the edge of the wound. Because MCF7 cells migrate much more slowly than HeLa (5 versus 22 μm/h), cells were fixed and stained for fluorescence microscopy at different time points (7 h for HeLa and 24 h for MCF7) that corresponded to an approximate 30% closing of the wound. As anticipated, the β3-producing cells in both cell lines became enriched at the edge of the wound (Supplementary Figure 1A). To quantify the results, β3 positive and negative cells at the edge of the wound were counted in 20 microscopic fields chosen at random in each of 3 independent experiments. The data confirmed that there was an increased percentage of β3-producing cells at the leading edge of the wound when paclitaxel was present (Supplementary Figure 1B) and indicated that β3 expression could be playing a significant role in the inability of microtubule targeted drugs to halt the migration of aggressive tumor cells.

### Resistance to paclitaxel inhibition of motility is due to β3-tubulin expression

**Figure 2:**
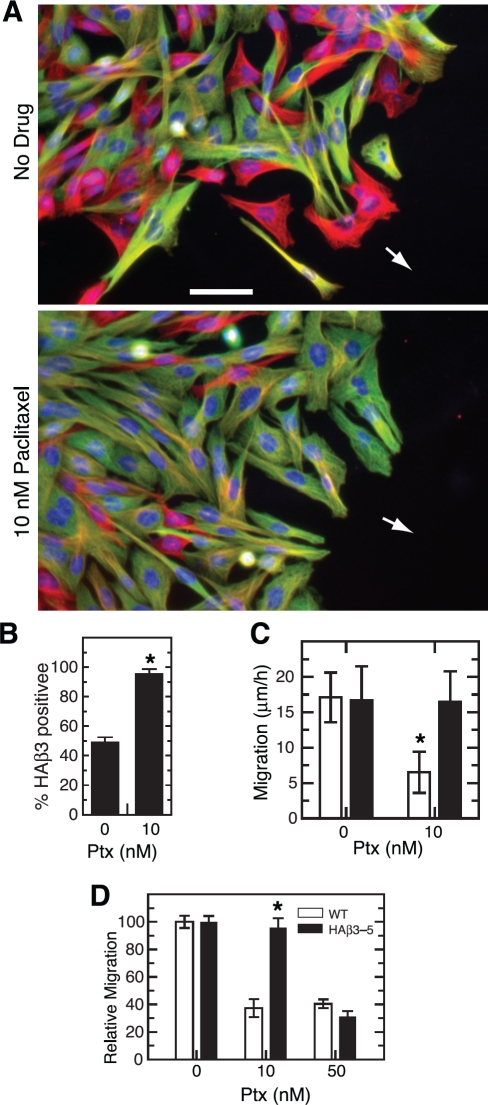
Paclitaxel inhibition of cell migration (A) A mixed culture of wild-type CHO cells and HAβ3-5 cells was scratched to make a wound and the cells were allowed to migrate for 24 h in the absence or presence of 10 nM paclitaxel. The cells were then stained for α-tubulin (red), HAβ3-tubulin (green), and DNA (blue). Arrows indicate the direction of movement. Bar, 50 μm. (B) Cells at the edge of the wound were scored for the presence or absence of HAβ3 expression and the percentage of HAβ3 containing cells was calculated from 20 random fields each containing approximately 20 cells along the leading edge. The experiment was repeated 3 times. (C) In a separate experiment, a pure culture was used to measure the rate of HAβ3-5 cell migration into a scratch wound with and without 10 nM paclitaxel in the presence (open bar) and absence (solid bar) of tetracycline. (D) A transwell assay was used to measure the migration of cells from the upper to the lower chamber over a 6 h period at various paclitaxel concentrations. The graph was generated by defining the number of cells that migrated to the lower chamber at 0 nM paclitaxel as 100% and expressing the data for the other concentrations relative to the zero control. Error bars, SD. *p < 0.05 relative to the WT control was considered significant.

Although the production of β3-tubulin appeared to correlate with the insensitivity of cell migration to paclitaxel suppression, it did not establish a cause and effect relationship. Human tumor cell lines are notoriously heterogeneous and the cells frequently differ by multiple genetic changes that could influence their response to paclitaxel treatment. Also, the cells were not uniform in their level of β3 production (see Figure 1B). Thus, cells with low production that might be sufficient to provide resistance could potentially be scored as β3-negative and thereby complicate the quantification of experiments such as those shown in Supplementary Figure 1. To eliminate these ambiguities, we turned to HAβ3-5, a cloned CHO cell line, created by transfection with HA-tagged β3-tubulin under the control of a tetracycline regulated promoter, that exhibits homogenous expression of the β3 isotype when the cells are grown in the absence of the antibiotic [15]. The parental CHO cells used to generate this cell line were shown to have no detectable expression of β3 by antibody binding (Figure 1A), 2D gel analysis [23], or sequencing of expressed cDNAs for β-tubulin [22]. By combining HAβ3-5 with wild-type cells, we created a wound healing experiment similar to that shown in Supplementary Figure 1A but with cells that were more clearly positive or negative for β3 production. For inhibition we used 10 nM paclitaxel, a concentration that is 1/5^th^ of the IC_50_ for inhibition of cell division in CHO cells [24]. The results showed that HAβ3-containing cells (green cells, Figure 2A) were relatively insensitive to the inhibitory effects of paclitaxel on cell migration and accumulated at the leading edge of the wound where they made up 95% of the cells versus only 50% of cells that migrated to the edge in the absence of drug (Figure 2B). As a further demonstration that β3 was the factor that mitigated the inhibitory effects of paclitaxel on cell migration, we directly measured the rates of migration of HAβ3-5 cells in the presence and absence of 10 nM paclitaxel under conditions in which HAβ3 was expressed or not expressed. The results showed that there was a significant paclitaxel-induced decrease in cell migration when the cells were tested in the presence of tetracycline (no HAβ3 expression; open bars in Figure 2C), but the decrease in migration did not occur when the cells were tested in the absence of tetracycline (with HAβ3 expression; solid bars in Figure 2C).

β3-Tubulin's ability to block paclitaxel inhibition of cell movement was also tested using a transwell migration assay in which wild-type or HAβ3-5 cells were plated on top of the membrane in the presence or absence of paclitaxel. After 6 h the membrane was fixed and stained with DAPI, and the cells that migrated to the bottom of the membrane were counted. It was observed that 10 nM paclitaxel effectively inhibited the migration of wild-type cells though the pores of the transwell membrane; but the migration of HAβ3-5 was not inhibited at this concentration (Figure 2D). At the toxic concentration of 50 nM paclitaxel, on the other hand, the migration of both cell lines was similarly inhibited. Thus, by two independent assays, β3-tubulin expression was able to counteract the inhibitory effects of 10 nM paclitaxel on cell migration.

### Paclitaxel inhibition of cell migration is mediated by suppression of microtubule dynamics

**Figure 3:**
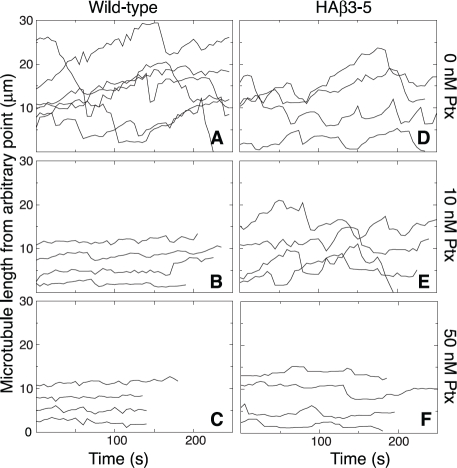
Paclitaxel suppression of microtubule dynamics Wild-type and HAβ3-5 cells were transfected with EGFP-MAP4 and microtubules were imaged every 5 s in the presence of the indicated concentrations of paclitaxel (Ptx). Microtubule lengths from an arbitrary internal reference point were plotted against time to describe the growth and shortening of the microtubule plus end. Each line represents a separate microtubule. Note that the position of the line on the y-axis is arbitrary and does not represent the actual total length of the microtubule.

We previously reported that low nontoxic concentrations of vinblastine, colcemid, and other microtubule disrupting drugs effectively suppressed microtubule dynamics and inhibited cell migration [20]. To determine whether the microtubule stabilizing drug paclitaxel also suppresses dynamics at low subtoxic concentrations, we generated microtubule life history plots showing time-dependent changes of microtubule length in the presence and absence of paclitaxel (Figure 3). In the absence of drug, microtubule plus ends in wild-type CHO cells exhibited frequent episodes of growth and shortening interspersed with periods of pause in which there was no significant change in length (Figure 3A). The addition of 10 nM paclitaxel (Figure 3B), a concentration that effectively inhibited cell migration but not cell division, caused a pronounced suppression of microtubule plus end excursions that was similar to the suppression caused by 50 nM drug (Figure 3C), the IC_50_ for inhibition of cell division [24]. Thus, paclitaxel, like vinblastine and colcemid, was able to suppress microtubule dynamics at low, subtoxic concentrations.

As previously reported [21], microtubules in HAβ3-5 cells were also dynamic in the absence of drug (Figure 3D); but unlike wild-type cells, the dynamics were not suppressed by 10 nM paclitaxel (Figure 3E). Instead, a much higher drug concentration (50 nM) was needed to cause suppression (Figure 3F). Drug-induced changes in the various parameters that describe dynamic instability such as growth and shortening rates are summarized in Supplementary Tables 1 and 2 for both of these cell lines. The ability of drugs that promote or inhibit microtubule assembly to decrease cell migration at the low concentrations that suppress microtubule dynamics supports the conclusion that dynamic microtubules are needed for cell movement. This conclusion is further supported by the observation that β3-tubulin prevented the suppression of both microtubule dynamics and cell migration that is normally caused by treatment with 10 nM paclitaxel.

**Figure 4:**
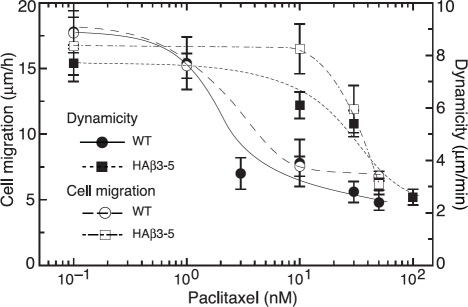
Effect of β3-tubulin expression on microtubule dynamics and cell migration Wild-type CHO cells (WT, circles) and HAβ3-5 cells (squares) were treated with varying concentrations of paclitaxel and the effects of the drug on microtubule dynamicity (solid symbols) and cell migration (open symbols) were measured. Note the relative resistance of both microtubule dynamics and cell migration to the inhibitory effects of paclitaxel in HAβ3-5 cells.

To further illustrate the relationship between microtubule dynamics and cell movement, we compared the rate of cell migration with dynamicity, a parameter used to characterize the extent of dynamic behavior [25], at several additional paclitaxel concentrations and plotted the results in Figure 4. A close correlation was found between paclitaxel suppression of dynamicity and inhibition of cell migration for both the wild-type and HAβ3-5 cells. Moreover, the drug concentration needed to inhibit both processes in HAβ3-5 was approximately 10-fold higher than in the wild-type cells.

### Paclitaxel increases pauses between cell movements

**Figure 5:**
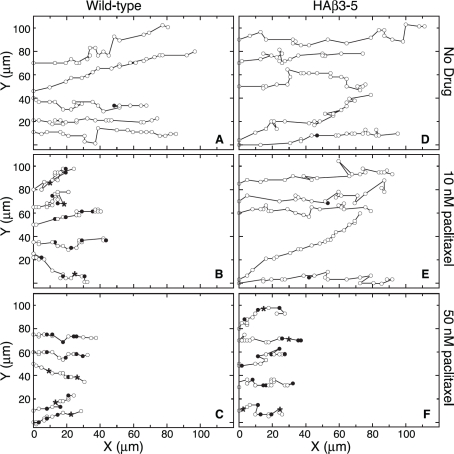
Paclitaxel effects on cell movement Wild-type and HAβ3-5 cells migrating into a wound in the presence and absence of paclitaxel were tracked for 5 h by marking the X and Y positions of the nucleus relative to a fixed internal reference point at 15 min intervals (open circles). For cases when the cell was paused (no movement) during one 15 min interval, the position is marked with a closed circle. When there was no movement during two 15 min intervals, the position is marked with an asterisk. Note that directionality didn't change, but the frequency of pauses greatly increased at inhibitory concentrations of paclitaxel.

**Figure 6:**
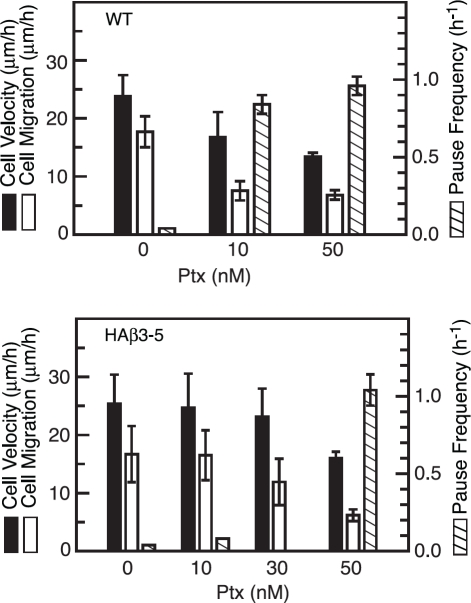
Calculation of cell movement parameters Microtubule tracks similar to those in Figure 5 were used to measure cell velocity (using only intervals during which the cells actually moved, solid bars), cell migration (1/2 the rate at which the wound closed, open bars), and frequency of pauses (number of intervals/h in which there was no cell movement, shaded bars).

To determine whether paclitaxel inhibited the rate of cell movement or simply altered the directionality of movement into the wound, we tracked individual cells every 15 min as they moved into a scratch wound and the results are summarized in Figure 5. In the absence of drug, wild-type and HAβ3-5 cells moved predominantly in the direction of the wound with relatively few excursions in other directions (Figure 5A). The addition of 10 nM paclitaxel did not alter the directionality of movement, but the progress of the wild-type cells in the direction of the wound was clearly inhibited (Figure 5B). Some of this inhibition was due to a smaller average distance moved during each 15 min interval reflecting a slower velocity of movement; but even more strikingly, there was an increased frequency of pauses in which the cells failed to move at all during one (solid circles) and sometimes two (asterisks) 15 min intervals. There was little further inhibition of cell movement when the paclitaxel concentration was increased to 50 nM (Figure 5C). Similar behavior was observed for cell line HAβ3-5, but inhibition of migration required the higher 50 nM drug concentration (Figure 5 D-F). The average velocities and pause frequencies for each cell line with increasing concentrations of paclitaxel are summarized in Figure 6. Paclitaxel caused relatively small decreases in the velocity of cell movement (dark bars, averaged only during the intervals the cells actually moved), but caused larger decreases in the rate of cell migration (light bars, calculated as ½ of the rate of wound closure). The difference between these two calculations could be explained by the large drug-induced increase in pauses during which the cells did not exhibit appreciable movement (shaded bars). These observations led us to conclude that paclitaxel acts primarily to prevent cell movement, and that it has quantitatively smaller effects on the directionality or velocity of cells in motion.

## DISCUSSION

Drugs that target mitosis have proven to be very effective agents for treating cancer. Most of these drugs bind directly to tubulin to either inhibit or promote microtubule assembly. The antitumor effects of these agents are usually attributed to direct killing of the tumor cells by activation of a mitotic checkpoint followed by apoptosis, but there is also evidence that antimitotic drugs are very effective inhibitors of angiogenesis and may thereby additionally act to limit tumor growth (reviewed in [26]). The mechanism by which microtubule targeted drugs inhibit mitosis and angiogenesis has been thought to involve the ability of these agents to suppress microtubule dynamics [27]. However, we recently demonstrated that low vinblastine and colcemid concentrations that were insufficient to inhibit cell division were nonetheless fully able to suppress microtubule dynamics, arguing that the drugs do not inhibit mitosis simply by suppressing this activity [20]. In contrast to the absence of effects on mitotic progression, suppression of microtubule dynamics had clear effects on cell migration. Subtoxic drug concentrations have previously been reported to inhibit cell motility [28-30], and our own studies recently showed that low, subtoxic concentrations of vinblastine, colcemid, and other drugs that affect microtubule assembly are able to inhibit cell migration and suppress microtubule dynamics with similar dose-response profiles [20]. In the studies described here, we demonstrated that the microtubule stabilizing drug paclitaxel acts in a similar manner.

Although there appears to be a close correlation between the ability of microtubule targeted drugs to suppress microtubule dynamics and their ability to inhibit cell migration, there is as yet no direct evidence that microtubule dynamics are required for cell motility. Previous studies, however, indicated that incorporation of β3-tubulin into microtubules reduced the effect of paclitaxel on dynamic instability [21]. If cell migration requires that microtubules be dynamic, we reasoned that β3 expression should also cause cell migration to be relatively “resistant” to the effects of the drug. The results described here confirmed that hypothesis; the dose responses for paclitaxel suppression of microtubule dynamics and inhibition of cell migration were both similarly shifted to approximately 10-fold higher drug concentrations when β3-tubulin was present. This outcome provides the strongest evidence to date that dynamic microtubules are required for cell motility.

The mechanism by which microtubule dynamics affect cell migration is unclear. Cells initiate motility by extending a lamellipodium at their leading edge in an actin-dependent manner, thereby causing them to stretch in the direction of movement [18]. The trailing edge must then release from the substratum and snap forward in order for the cell to translocate, and there is evidence to suggest that microtubules control this latter process [31]. In agreement, we recently reported that suppressing microtubule dynamics with vinblastine did not stop migrating cells from extending a lamellipodium but rather appeared to inhibit the ability of the tail to retract [20]. These observations are consistent with the discovery that microtubules are stabilized at the leading edge of migrating cells but remain dynamic at the trailing edge [32]. Microtubule involvement in tail retraction could be mediated by effects on adhesion site turnover, or by effects on the actin cytoskeleton, but exactly how microtubules are involved remains an unsettled question [19]. Our data demonstrate that microtubule dynamics are critical for their ability to influence cell movement.

Our results also have important clinical implications. A number of studies have noted that tumors from diverse tissues inappropriately express β3-tubulin, an isoform that is normally restricted to brain and Sertoli cells [1, 2]. Moreover, β3 is usually seen in tumors that are especially difficult to treat and it frequently is found in tumors from patients who have relapsed [16, 17, 33]. Again, however, these observations represent correlations and there is little evidence to suggest that β3 plays a direct role in the response to treatment. It is likely that during their growth tumor cells have accumulated many mutations and other changes that could affect their behavior. Thus, the appearance of a protein such as β3 might, or might not, have functional consequences. In this regard, it was recently shown that β3 is induced in tumors that have become hypoxic and/or starved for glucose [34, 35]. As such, it might be only one of a series of genes that have been activated due to the conditions under which the tumor cells are growing, and could be a marker for aggressiveness rather than a driver of the behavior.

Arguing in favor of a direct role of β3 in tumor cell behavior, a number of studies have reported that cells selected for resistance to the cytotoxic effects of paclitaxel have increased expression of β3, suggesting that this isotype can confer resistance to the drug [13, 14]. Subsequent studies in our laboratory confirmed this idea but demonstrated that increased β3 expression alone can only confer very weak 1.5-fold resistance and that it acts by altering the extent of microtubule assembly [15]. In contrast to the weak effects on the cytotoxic action of the drug, we show here that β3 expression produces a 10-fold resistance to the ability of paclitaxel to inhibit cell migration. Because cell migration is a critical step in angiogenesis and tumor cell metastasis, our data suggest that β3's effects on motility are a better explanation for the observation that its expression correlates with poor prognosis.

## METHODS

### Cell lines

Chinese hamster ovary (CHO) cells were grown at 37 °C and 5% CO_2_ in α-MEM supplemented with 5% fetal bovine serum, 50 U/ml penicillin, and 50 μg/ml streptomycin. HAβ3-5, a CHO cell line that expresses HA-tagged β3-tubulin under the control of a tetracycline (tet) regulated promoter [15], was maintained in the presence of tet; β3-tubulin expression was induced by growing the cells overnight in medium without tet. Human cancer cell lines including HeLa, DU145, MCF7, K562, and KB3 were grown in α-MEM with 10% fetal bovine serum.

### Immunofluorescence

Cells on sterile glass coverslips were fixed in methanol at –20 °C, or in some cases, they were pre-extracted before fixation by incubating the coverslips for 1 min at 4 °C in microtubule stabilizing buffer (20 mM Tris-HCl (pH 6.8), 1 mM MgCl_2_, 2 mM EGTA, 0.5% NP40) containing 4 μM paclitaxel. Following fixation, the cells were rehydrated in PBS and incubated with a 1:100 dilutions of mouse monoclonal antibody TUJ1 (Covance, Princeton, NJ) (to detect β3-tubulin) and rabbit polyclonal antibody X2 (gift of Dr. Chloe Bulinski, Columbia University) (to detect α-tubulin) for 2 h at 37 °C. The HA tagged β3-tubulin overexpressing CHO cells were stained with similar dilutions of rabbit polyclonal HA antibody (Bethyl Labs, Montgomery, TX) and DM1A (Sigma-Aldrich, St. Louis, MO), a monoclonal antibody to detect α-tubulin. After washing, the cells were further stained for 1 h with a 1:100 dilutions of Alexa 488-conjugated goat anti mouse IgG and Alexa 594-conjugated goat anti rabbit IgG (Invitrogen) that included 1 μg/ml 4',6-diamidino-2-phenylindole (DAPI) to stain DNA. Cells were viewed using an Optiphot microscope (Nikon Inc., Melville, NY) equipped with a MagnaFire digital camera (Optronics, Goleta, CA).

### Electrophoresis and western blots

Cellular proteins were solubilized in SDS sample buffer, separated on 7.5% polyacrylamide minigels, and transferred to nitrocellulose membranes. The membranes were incubated with 1:2,000 dilutions of TUJ1 and actin antibody C4 (Chemicon, Temecula, CA) followed by Alexa 647-conjugated goat anti-mouse IgG (Invitrogen). Bands were detected by fluorescence emission using a Storm 860 scanner (GE Healthcare).

### Cell migration assay

Cells were grown in a monolayer to about 85% of confluence. A scratch was made at the center of the petridish using a wooden toothpick, and fresh medium was added with or without paclitaxel. Wound closure was monitored using an inverted microscope and 4X phase objective. The wound was photographed every 15 minutes for 8 h and the rate of wound closure was measured from the slope of the gap size plotted against time. This value was divided by 2 to arrive at the rate of cell migration at each edge of the wound.

### Transwell assay

Approximately 5000 cells were seeded into the upper chamber of 24-well cell culture inserts containing 8 μm membrane pores (BD Biosciences) and allowed to settle for 2 h in αMEM containing 10% FBS. Medium was then removed and replaced with fresh medium containing varying concentrations of paclitaxel. The dishes were incubated a further 6 h to allow cell migration, and residual cells in the upper chamber were removed with a cotton swab. The membranes were then excised, fixed in methanol, and stained with DAPI. The nuclei on the bottom membrane surface were counted from 20 microscopic fields chosen at random using a fluorescence microscope and 20X objective. A minimum of 300 cells were counted for each data point and the experiment was repeated twice.

### Transfection and live cell microscopy

Cells were seeded onto sterile 25 mm circular coverslips and transfected with EGFP-MAP4 (provided by Dr. Joanna Olmsted, University of Rochester) using Lipofectamine (Invitrogen). After transfection the cells were maintained in the presence or absence of paclitaxel for 2 d and then transferred to McCoy's 5A medium containing 25 mM HEPES (Mediatech) with the appropriate concentration of paclitaxel. Images were captured 5 s apart at 37 °C using a DeltaVision Core imaging system (Applied Precision Inc., Issaquah, WA) equipped with a CCD camera and imaging software supplied by the vendor.

### Calculating microtubule dynamics

The contour distance between the plus-end of a microtubule and an arbitrary reference point on the same microtubule was measured using ImageJ software and graphed as a function of time to generate life history plots. The rates of growth and shortening were calculated from the slopes of the plots using linear regression. If the change in length of a microtubule between two successive time points was greater than 0.5 μm, it was considered real growth or shortening; otherwise, it was treated as noise. Catastrophe was defined as the transition from either growth or pause to shortening, and the catastrophe frequency was calculated as the number of transitions divided by the time spent in growth and pause. Rescue was defined as the transition from shortening to either growth or pause, and the rescue frequency was calculated as the number of such events divided by the time spent shortening. Dynamicity, an overall measure for how dynamic the microtubules behave, was calculated by dividing the total change in length (including both growth and shortening) of a microtubule by the total time the microtubule was under observation. Each parameter was calculated from microtubules persisting for more than 2 min and is expressed as the mean ± standard error. The student t-test was used to compare parameters between different cell lines and treatments. Differences were considered significant when the p value was less than 0.05.

## 







## Abbreviations

CHO: Chinese hamster ovary
HA: hemagglutinin antigen
ptx: paclitaxel
tet: tetracycline
