# Supplementary material for: Class III β-Tubulin Counteracts the Ability of Paclitaxel to Inhibit Cell Migration
Source: Oncotarget. 2011 May 16;2(5):368–77. doi: 10.18632/oncotarget.250 (PMC3248193; doi:10.18632/oncotarget.250)
Supplement: Supplementary file 2 [file oncotarget-02-368-s002.doc]

**Supplementary Table 1:** Effect of paclitaxel on microtubule dynamics in wild-type CHO cells

——————————————————————————————————————————————

nM Paclitaxel 0 1 3 10 30 50

——————————————————————————————————————————————

Growth

Rate (μm/min) 16.1 ± 1.2 13.1 ± 1.3* 9.9 ± 0.8* 9.9 ± 0.6* 10.7 ± 0.5* 9.5 ± 0.8*

Duration (s) 11.0 ± 1.0 7.6 ± 0.4* 6.6 ± 0.7* 6.2 ± 0.4* 7.6 ± 0.9* 5.8 ± 0.6*

Distance (μm) 2.6 ± 0.2 1.7 ± 0.2* 1.1 ± 0.2* 1.0 ± 0.1* 1.3 ± 0.2* 0.9 ± 0.1*

Shortening

Rate (μm/min) 28.2 ± 4.1 20.4 ± 1.6* 13.4 ± 1.0* 12.1 ± 1.3* 11.2 ± 0.7* 9.3 ± 0.5*

Duration (s) 11.6 ± 1.6 8.3 ± 0.8* 6.0 ± 0.4* 7.3 ± 0.9* 6.3 ± 0.5* 5.3 ± 0.3*

Distance (μm) 4.4 ± 0.6 2.7 ± 0.3* 1.3 ± 0.1* 1.7 ± 0.4* 1.3 ± 0.2* 0.8 ± 0.1*

% Time

Growth 30.5 ± 2.3 21.9 ± 1.8* 14.7 ± 2.3* 16.0 ± 3.2* 14.5 ± 2.6* 9.8 ± 1.5*

Shortening 20.9 ± 1.8 23.9 ± 3.2 15.4 ± 2.7 14.7 ± 2.3 10.6 ± 2.0* 12.1 ± 2.6*

Pause 48.6 ± 4.3 54.3 ± 4.4 69.9 ± 4.5* 69.3 ± 5.2* 74.9 ± 3.1* 78.2 ± 3.6*

Frequency (min^-1^)

Catastrophe 1.5 ± 0.2 2.5 ± 0.4* 1.8 ± 0.3 1.6 ± 0.2 1.3 ± 0.2 1.6 ± 0.3

Rescue 6.3 ± 0.8 7.3 ± 0.5 10.2 ± 0.8* 7.9 ± 1.0 8.8 ± 0.9* 9.9 ± 1.2*

Dynamicity (μm/min) 8.9 ± 0.8 7.6 ± 1.1 3.6 ± 0.7* 3.8 ± 0.9* 2.7 ± 0.5* 2.4 ± 0.3*

———————————————————————————————————————————————

At least 15 microtubules were analyzed at each concentration.

Values represent the mean ± sem

*p<0.05 when compared to CHO WT
