# Supplementary material for: Class III β-Tubulin Counteracts the Ability of Paclitaxel to Inhibit Cell Migration
Source: Oncotarget. 2011 May 16;2(5):368–77. doi: 10.18632/oncotarget.250 (PMC3248193; doi:10.18632/oncotarget.250)
Supplement: Supplementary file 3 [file oncotarget-02-368-s003.doc]

**Supplementary Table 2:** Effect of paclitaxel on microtubule dynamics in HAβ3-5 cells

——————————————————————————————————————————————

nM Paclitaxel 0 10 30 50 100

——————————————————————————————————————————————

Growth

Rate (μm/min) 12.5 ± 0.7* 12.1 ± 0.5* 11.4 ± 0.5* 9.2 ± 0.5* 8.5 ± 0.6*

Duration (s) 9.4 ± 1.2 7.2 ± 0.4* 7.7 ± 0.6* 6.5 ± 0.6* 6.1 ± 0.3*

Distance (μm) 2.0 ± 0.4 1.5 ± 0.1* 1.5 ± 0.2* 1.0 ± 0.1* 0.8 ± 0.1*

Shortening

Rate (μm) 19.8 ± 1.6 23.2 ± 4.2 18.4 ± 1.0* 14.4 ± 1.2* 10.3 ± 0.6*

Duration (s) 8.7 ± 0.5 8.5 ± 0.5 8.4 ± 0.6 6.5 ± 0.5* 6.2 ± 0.3*

Distance (μm/min) 2.9 ± 0.3* 3.0 ± 0.3 2.5 ± 0.2* 1.6 ± 0.2* 1.1 ± 0.1*

% Time

Growth 25.9 ± 1.7 17.8 ± 2.6 20.4 ± 1.8 11.2 ± 1.0* 11.8 ± 1.4*

Shortening 21.5 ± 1.4 20.4 ± 2.2 16.5 ± 1.5 13.8 ± 1.6 15.4 ± 2.0

Pause 52.6 ± 2.7 61.8 ± 3.0 63.1 ± 2.6 74.9 ± 2.2* 72.7 ± 2.5*

Frequency (min^-1^)

Catastrophe 2.0 ± 0.2* 1.9 ± 0.2 1.5 ± 0.2 1.5 ± 0.2 1.8 ± 0.3

Rescue 6.3 ± 0.5 6.8 ± 0.6 7.1 ± 0.5 9.3 ± 0.7* 9.6 ± 0.4*

Dynamicity (μm/min) 7.7 ± 0.7 6.1 ± 0.5* 5.4 ± 0.5* 3.0 ± 0.3* 2.6 ± 0.3*

———————————————————————————————————————————————

At least 15 microtubules were analyzed at each concentration.

Values represent the mean ± sem

*p<0.05 when compared to CHO WT
